# Determining a methodology of dosimetric quality assurance for commercially available accelerator-based boron neutron capture therapy system

**DOI:** 10.1093/jrr/rrac030

**Published:** 2022-06-20

**Authors:** Katsumi Hirose, Takahiro Kato, Takaomi Harada, Tomoaki Motoyanagi, Hiroki Tanaka, Akihiko Takeuchi, Ryohei Kato, Shinya Komori, Yuhei Yamazaki, Kazuhiro Arai, Noriyuki Kadoya, Mariko Sato, Yoshihiro Takai

**Affiliations:** Department of Radiation Oncology, Southern Tohoku BNCT Research Center and Southern Tohoku General Hospital, 7-10 Yatsuyamada, Koriyama, Fukushima 963-8052, Japan; Department of Radiology and Radiation Oncology, Hirosaki University Graduate School of Medicine, 5 Zaifu-cho, Hirosaki, Aomori 036-8562, Japan; Department of Radiation Oncology, Southern Tohoku BNCT Research Center and Southern Tohoku General Hospital, 7-10 Yatsuyamada, Koriyama, Fukushima 963-8052, Japan; Department of Radiation Oncology, Southern Tohoku Proton Therapy Center, 7-172 Yatsuyamada, Koriyama, Fukushima 963-8052, Japan; School of Health Sciences, Fukushima Medical University, 10-6 Sakaemachi, Fukushima 960-8516, Japan; Department of Radiation Oncology, Southern Tohoku BNCT Research Center and Southern Tohoku General Hospital, 7-10 Yatsuyamada, Koriyama, Fukushima 963-8052, Japan; Department of Radiation Oncology, Southern Tohoku BNCT Research Center and Southern Tohoku General Hospital, 7-10 Yatsuyamada, Koriyama, Fukushima 963-8052, Japan; Particle Radiation Oncology Research Center, Institute for Integrated Radiation and Nuclear Science, Kyoto University, 2 Asashiro-nisi, Sennan-gun, Osaka 590-0494, Japan; Department of Radiation Oncology, Southern Tohoku BNCT Research Center and Southern Tohoku General Hospital, 7-10 Yatsuyamada, Koriyama, Fukushima 963-8052, Japan; Department of Radiation Oncology, Southern Tohoku BNCT Research Center and Southern Tohoku General Hospital, 7-10 Yatsuyamada, Koriyama, Fukushima 963-8052, Japan; Department of Radiation Oncology, Southern Tohoku BNCT Research Center and Southern Tohoku General Hospital, 7-10 Yatsuyamada, Koriyama, Fukushima 963-8052, Japan; Department of Radiation Oncology, Southern Tohoku BNCT Research Center and Southern Tohoku General Hospital, 7-10 Yatsuyamada, Koriyama, Fukushima 963-8052, Japan; Department of Radiation Oncology, Southern Tohoku BNCT Research Center and Southern Tohoku General Hospital, 7-10 Yatsuyamada, Koriyama, Fukushima 963-8052, Japan; Department of Radiation Oncology, Southern Tohoku Proton Therapy Center, 7-172 Yatsuyamada, Koriyama, Fukushima 963-8052, Japan; Department of Radiation Oncology, Tohoku University School of Medicine, 1-1 Seiryo-machi, Aoba-ku, Sendai 980-8574, Japan; Department of Radiation Oncology, Southern Tohoku BNCT Research Center and Southern Tohoku General Hospital, 7-10 Yatsuyamada, Koriyama, Fukushima 963-8052, Japan; Department of Radiology and Radiation Oncology, Hirosaki University Graduate School of Medicine, 5 Zaifu-cho, Hirosaki, Aomori 036-8562, Japan; Department of Radiation Oncology, Southern Tohoku BNCT Research Center and Southern Tohoku General Hospital, 7-10 Yatsuyamada, Koriyama, Fukushima 963-8052, Japan

**Keywords:** accelerator-based boron neutron capture therapy, dosimetric quality assurance

## Abstract

The irradiation field of boron neutron capture therapy (BNCT) consists of multiple dose components including thermal, epithermal and fast neutron, and gamma. The objective of this work was to establish a methodology of dosimetric quality assurance (QA), using the most standard and reliable measurement methods, and to determine tolerance level for each QA measurement for a commercially available accelerator-based BNCT system. In order to establish a system of dosimetric QA suitable for BNCT, the following steps were taken. First, standard measurement points based on tissue-administered doses in BNCT for brain tumors were defined, and clinical tolerances of dosimetric QA measurements were derived from the contribution to total tissue relative biological effectiveness factor-weighted dose for each dose component. Next, a QA program was proposed based on TG-142 and TG-198, and confirmed that it could be assessed whether constancy of each dose component was assured within the limits of tolerances or not by measurements of the proposed QA program. Finally, the validity of the BNCT QA program as an evaluation system was confirmed in a demonstration experiment for long-term measurement over 1 year. These results offer an easy, reliable QA method that is clinically applicable with dosimetric validity for the mixed irradiation field of accelerator-based BNCT.

## INTRODUCTION

Clinical studies of boron neutron capture therapy (BNCT) have been carried out using thermal and epithermal neutron generated from nuclear reactors over several decades since the theory of NCT was proposed by Locher in 1936 [1]. To date, several BNCT studies have been performed for patients with refractory cancers such as glioblastoma and recurrent head and neck cancer, and preferable treatment outcomes have been achieved even for the patients with no treatment options using existing radiotherapy modalities due to limit of the tolerable dose to organs-at-risk [2–26]. In recurrent glioma with WHO grade III and IV, BNCT is suggested to contribute to improvements in overall survival (OS), especially in the group with RPA class III or VII, which has a worse prognosis than the others [3]. Likewise, in newly diagnosed glioblastoma patients, compared to the Stupp regimen of standard therapy, BNCT combined with radiotherapy with 20–30 Gy is reported to attain better OS [4, 5, 27, 28]. For head and neck cancer, patients already irradiated with 63–165 Gy can receive BNCT treatment with no severe adverse effects and acquisition of better tumor response [12]. However, almost previous studies have been based on reactor use.

The first commercial accelerator-based BNCT system, NeuCure (Sumitomo Heavy Industries, Ltd., Tokyo, Japan) was developed as joint research in collaboration with Kyoto University and Sumitomo Heavy Industries, Ltd. In this system, protons, converted from negative hydrogen ions accelerated by a 30-MeV accelerator through charge converting foil, collide with a beryllium target to generate neutrons with broad energy, which are decelerated by a moderator composed of lead, iron and aluminum, and irradiated to the patient as epithermal neutrons. One of the advantages of this accelerator-based epithermal neutron generator is that a constant beam output can be stably obtained, regardless of the date and time under adequate quality assurance (QA) and quality control (QC), and linearity between the current value of the accelerator to be used and the neutron flux is expected [29, 30]. Thus, appropriate QA procedures for an accelerator-based epithermal neutron generator may eliminate the need for measurement of neutron flux during each treatment, which must be performed during treatment with the nuclear reactor [31]. Although much work on beam modeling and developing real-time beam detectors for BNCT has been undertaken, insufficient evidence has been accumulated regarding dosimetric QA procedures for clinical use [32]. The few reports on QA procedures are all related to the constancy of reactor-based neutron sources and do not go far enough to the dosimetric accuracy that should be required for conventional radiotherapy. This is because it is very time consuming to measure neutron flux based on the metal activation method, which is used as the most basic and standard one which are used as a reference also in the study and development of the other novel neutron detectors [33–43]. Although combinations of a few of neutron detectors and ion chambers have also been used for dosimetry in nuclear reactors, which may be useful in simplifying the process, there has been no discussion on whether adequate sensitivity and accuracy of these instruments are provided, and whether reasonable tolerance levels can be set based on them. In addition, due to the characteristics of these measuring instruments, a decrease in sensitivity over time may be inevitable, so it is necessary to introduce a correction factor for the measured values, and eventually it is necessary to guarantee the quantitativeness of the measured values by frequent metal-based activation methods. Before discussing the applicability of these devices to QA, it is necessary to discuss what degree of dosimetric uncertainty is acceptable to ensure clinically necessary dosimetric accuracy in the irradiation fields used for BNCT, and the requirements for measurement devices such as real-time monitors and ion chambers will be clarified as the next step.

The goal of this work was to establish methodology of dosimetric QA for accelerator-based BNCT to achieve reasonable accuracy with workload volume suitable for clinical practice in medical institutions. In this report, we propose a methodology for dosimetric QA of BNCT treatment devices considering the contribution proportion by relative biological effectiveness factor (RBE)-weighted dose. We first optimized the QA procedure for accelerator-based BNCT with reference to QA for linear accelerator especially based on TG-142 and TG-198 from American Association of Physics in Medicine (AAPM) [44, 45]. In the process, a comparison with the detailed description of dosimetric QA for the linear accelerator was performed to validate the procedure.

## MATERIALS AND METHODS

### General concept of determining dosimetric QA program in accelerator-based BNCT for clinical use

The irradiation field of BNCT consists of neutrons with a broad energy spectrum including thermal, epithermal and fast neutron, and gamma. The contribution of each component varies from site to site, such that a dominant component at one site may less contribute at the other site so that it is difficult to measure with sufficient measurement precision using standard measurement methods. The metal activation is the most fundamental method of neutron measurement, but it is with relatively large measurement dispersion. Due to these small component contributions and measurement dispersion from site to site, it is impossible to guarantee sufficient dosimetric constancy even if the QA program of BNCT is constructed in the same way as the QA program of linear accelerator for each component. Moreover, a QA program constructed in this way would require a huge amount of work, unnecessarily consuming a vast amount of electric power, and needlessly exhausting the BNCT system.

In order to establish a system of dosimetric QA suitable for BNCT, we took the following steps: (i) We set up standard measurement points based on tissue-administered RBE-weighted doses in BNCT for brain tumors, and derived tolerance levels of dosimetric QA for each dose component at each point from clinical aspect (i.e. clinical tolerances); (ii) we proposed a QA program based on TG-142 and TG-198 [44, 45], and confirmed that it could be assessed whether constancy of each dose component was assured within the limits of determined tolerances or not by measurements of the proposed QA program; and (iii) the validity of the BNCT QA program as an evaluation system was confirmed in a demonstration experiment for long-term measurement over 1 year with the QA program being updated gradually.

### Establishment of standard measurement points and clinical tolerances

A 200-mm cubic water phantom which is considered to be closer to tissues than resins such as PMMA was used as the measurement system. The representative standard measurement points were determined as reference points (RPs) from the critical points on the dose distribution on the cubic phantom used for QA measurements, which is given when the phantom is regarded as human tissues, such as tumor and normal brain tissue. Next, contribution of each dose component to the total RBE-weighted tissue dose at each RP was clarified. From the contribution, the components that should be secured for dosimetry at each RP were extracted.

Calculation of tumor and normal brain doses was performed with the BNCT treatment planning system, SERA [46], assuming a blood boron concentration of 25 ppm as an ordinary level and 3.5-fold accumulation of boron agent into tumors compared to blood, as well as assuming RBEs for nitrogen, hydrogen, and gamma rays as 2.9, 2.4, and 1.0, and the compound biological effectiveness factor (CBE) for ^10^B-boronophenylalanine (^10^B-BPA) as a boron agent for tumor as 4.0 and for normal brain tissue as 1.34 [47–50]. Specifically, after transportation calculation regarding the phantom as water, the boron dose, nitrogen dose, hydrogen dose and gamma dose on the beam axis in the phantom were derived by regarding the phantom as a single tissue, such as tumor tissue or normal brain tissue, to be evaluated and giving values for tissue composition, boron concentration and CBE. The various parameters used in the derivation are listed in Supplementary Table A1.

Our concept of dosimetric QA for BNCT, which involves difficulties in measuring dose components, was that the integrity of the device should be assured at least to the extent that it can guarantee clinically given effects under assumed tissue boron concentrations as much as possible. The clinical tolerances of dosimetric QA for each dose component was calculated as variability of single dose components allowed within 3% of total RBE-weighted dose. It should be noted that the additivity of the variance between dose components does not hold strictly because the production of them can affect each other. But if assuming it does hold, then the synthesized dosimetric deviation of total RBE-weighted dose is the root sum square of deviations of thermal neutron-dependent boron and nitrogen doses, and fast neutron-dependent hydrogen dose and gamma, as follows:

$$\begin{align*} & \Delta \mathrm{Total}\ \mathrm{RBE}\hbox{-}\mathrm{weighted}\ \mathrm{dose} \\ & =\sqrt{\begin{array}{@{}l} {\left(\Delta \mathrm{RBE}\hbox{-}{\mathrm{weighted}\ \mathrm{dose}}_{Thermal}\right)}^2 \\ \quad +{\left(\Delta \mathrm{RBE}\hbox{-}{\mathrm{weighted}\ \mathrm{dose}}_{Fast}\right)}^2 \\ \quad +{\left(\Delta \mathrm{Gamma}\ \mathrm{dose}\right)}^2 \end{array} } \end{align*}$$

When these deviations are all within ±3%, deviation of the total RBE-weighted dose is kept within ±5.2%.

### Development of a QA program optimized for accelerator-based BNCT

**Fig. 1:**
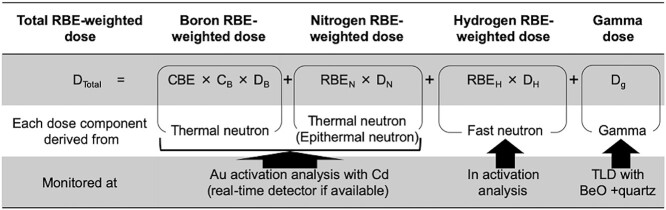
Scheme of dosimetry for cyclotron BNCT at medical institutions. Abbreviation: RBE, relative biological effectiveness factor; D_total_, Total RBE-weighted dose; CBE, compound biological effectiveness factor; C_B_, ^10^B concentration; D_B_, ^10^B neutron capture absorbed dose; RBE_N_, relative biological effectiveness factor for nitrogen; D_N_, nitrogen absorbed dose derived from ^14^N(n,p)^14^C reaction; RBE_H_, relative biological effectiveness factor for hydrogen; D_H_, hydrogen absorbed dose derived from H(n,p)H reaction; D_g_, gamma absorbed dose; Au, gold; In, indium; TLD, thermoluminescence dosimeter.

QA procedures were extracted from dosimetric QA summarized in AAPM TG-142 and TG-198, excluding items related to contents not considered in BNCT, such as rotating gantries, wedges and TBI/TSET. Each QA procedure consisted of evaluation of thermal and epithermal neutrons with bare and cadmium-covered gold foils or wires, fast neutrons with indium foils and gamma rays with thermoluminescence dosimeter (TLD), as shown in Fig. 1. The goal of the daily QA was set to be completed within 30 min, giving top priority to practicality. If the daily QA is insufficient to determine whether the dosimetric accuracy of all components is maintained, a plan to add weekly QA procedures that could supplement the daily QA was considered. For monthly QA and annual QA, consideration was given that the measurement items were composed only for dose components and measurement points for which sufficient measurement precision was maintained compared to the tolerance level. On the contrary, in the case of components and positions whose measurement precision is poor and not lead to the evaluation of dosimetric accuracy, the measured values are treated as references only, and it was planned to judge the accuracy together with that of other components. Or measurement was omitted if poor measurement precision is not commensurate with the workload.

### Measurement of thermal, epithermal and fast neutrons

For the measurement of thermal neutrons in the energy range below 0.5 eV, gold foils or wires available depending on the activation after irradiation at each point, and cadmium-covered gold wires in some cases, were placed at each point, including the specific RPs, and irradiated. For a single point measurement on the geometry, the irradiation and measurement were performed using 0.05-mm-thick gold foil (purity: 99.95%; AU-173261; Nilaco, Tokyo, Japan) with or without 0.7-mm-thick cadmium cover (purity: 99.9%; provided by Kyoto University Institute for Integrated Radiation and Nuclear Science [51]). On the other hand, in order to measure at multiple points on a straight line at once, a gold wire with a diameter of 0.25 mm (purity: 99.95%; Au-171 285; Nilaco) was arranged in a straight line and irradiated, then cut out in 5-mm lengths according to the measurement location, and formed into a spiral shape before measurement. In the case of calculating the thermal neutron flux, a separate gold wire was covered with a 0.5-mm-thick cadmium outer tube (purity: 99.95%; SWX-630; Bladewerx, New Mexico) and measured by the same procedure. Activity of gold was measured using a high-purity germanium detector (HP-Ge) (GEM20P4–70; ORTEC, Oak Ridge, TN, USA) and the thermal neutron flux was estimated from the cadmium ratio [31]. The reaction rate calculated from a net count of the activity of gold wires by HP-Ge, reflecting mainly thermal and epithermal neutrons, were adopted as alternatives to thermal and epithermal neutron flux. The reaction rate (RR) was calculated as follows.

$$ \mathrm{RR}\!=\!\frac{\lambda{C}}{\varepsilon \gamma{{N}}_0\left({{e}}^{-\lambda{{t}}_1}\!-\!{{e}}^{-\lambda{{t}}_2}\right){\sum}_{{i}=1}^{{n}}\left[\frac{{{Q}}_{{i}}}{\Delta{t}}\cdot \left({{e}}^{-\lambda \left({n}-{i}\right)\cdot \Delta{t}}-{{e}}^{-\lambda \left({n}-{i}+1\right)\cdot \Delta{t}}\right)\right]} $$

where the constants *λ*, *ε*, *γ*, *N_0_* and *C* are the decay constant, detection efficiency, gamma-ray emission ratio, total number of atoms in gold, and total photon-peak counts, respectively. Since RR is calculated as the value for operation at 1 mA in the accelerator, *Q_i_* is introduced as a correction value for the variation of the current value, and *n*·Δ*t* is irradiation time, and *t_1_* and *t_2_* are the times to start and end of measurement from the end of irradiation, respectively. The derivation of the formula was shown in the Supplementary Data.

The thermal neutron flux was calculated from the reaction rate for thermal neutrons alone, which was obtained by subtracting the reaction rate of gold covered with cadmium from that of without cadmium. In situations where the increased measurement uncertainty associated with two independent measurements requiring energy discrimination by the presence of cadmium cannot be tolerated, an evaluation using the active reaction rate itself was applied as an alternative.

For the measurement of fast neutrons, activities of 0.5-mm-thick indium foils (purity: 99.99%; In-203 321; Nilaco) after irradiation were examined based on the threshold of nuclear reaction energy at 1.2 MeV, following a previous study [51]. Briefly, a 336 keV photon emitted from ^115m^In, which was produced based on ^115^In(n,n’)^115m^In reaction by inelastic scattering with fast neutrons, was measured. ^115^In(n,n’)^115m^In reaction has a lower reaction threshold than others, and has the best threshold energy for fast neutron detection and is suitable for measurement. However, unnecessary reaction of ^115^In(n,γ)^116m^In, mainly due to capture reactions with thermal neutrons, results in multiple photon spectra containing high energies. For this reason, the foil was covered with 0.7-mm cadmium cover to suppress the unwanted reaction as much as possible. Due to the total fast neutron flux cannot be derived from actual measurements of the activation rate using an indium based on the threshold of nuclear reaction energy, an evaluation using the active reaction rate itself was assumed as an alternative.

### Measurement of gamma dose

It was taken into account that QA instrument tools for linac used for photon evaluation could not be used in the neutron field because neutron capture would cause instrument failure and production of long half-life nuclides. Therefore, TLD was used for measurement of gamma. As TLD elements, UD-170LS (BeO type, Matsushita Electric Co, Osaka, Japan) embedded in quartz was used. The main component of the hard glass tube covering the outside of UD-170 L is SiO_2_, but other substances such as B_2_O_3_, Na_2_O, Al_2_O_3_, which cause high sensitivity to thermal neutrons, are contained [52]. The UD-170LS, as a specially customized device in which the rigid glass capillary was replaced with a quartz glass tube and the fluorescent substance BeO resulting in reduction of sensitivity to thermal neutrons, was therefore adopted. According to our preliminary investigation, the thermal neutron sensitivity of UD-170LS is 4.0 ± 0.9 × 10^−13^ Gy cm^2^, which is about 17% of that of UD-170 L with 2.4 ± 0.2 × 10^−12^ Gy cm^2^. One hour after irradiating the mixed beam with a constant charge amount to these pre-annealed TLD elements arranged in each standard measurement point, TLD elements were analyzed using a TLD reader (UD-5120PGL; Matsushita Electric Co, Osaka, Japan) in 6 seconds at 420°C. The dose indicated from TLD was converted to the gamma dose rate (Gy/h) using the correction coefficient which was given for each element as the reciprocal of the value (in Sv) of TLD elements read by the TLD reader after 1.00 Gy of X-irradiation with a linear accelerator to TLD elements placed at a constant source-axis distance of 100 cm.

## RESULTS

### Determination of measurement points and clinical tolerances of dosimetric QA for each dose component from clinical aspect

**Fig. 2:**
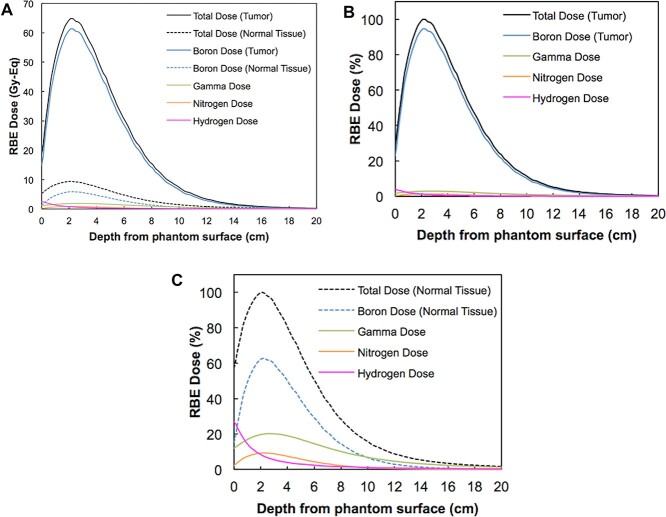
The absorbed dose components in phantom. Each RBE-weighted dose component was calculated from the simulated distribution of neutron flux and gamma dose rate with SERA multiplying the assumed RBEs and CBE for boronophenylalanine, as well as considering the assumed boron concentration of the tumor tissue. (A) Each RBE dose component for tumor and normal tissues are presented with Gy-Eq. (B) and (C) represent proportions of components in tumor and normal tissue, respectively. Abbreviations: RBE, relative biological effectiveness factor; CBE, compound biological effectiveness factor.

For tumor RBE-weighted dose in 200-mm cubic water phantom, obtained as a result of simulation by SERA, the boron dose depending on thermal neutron was dominant at all depths of the phantom. The fast neutron dose is subdominant at the phantom surface, occupying 13.3% as hydrogen dose of all doses, while the gamma dose was even less, at 5.9% (Fig. 2 and Table 1). At the peak tumor RBE-weighted dose occurring around 20 mm in depth, the thermal neutron dose was dominant at 95.8% as the total boron RBE-weighted and nitrogen RBE-weighted dose, and a 2.9% gamma dose and 1.2% fast neutron dose were included. The tumor RBE-weighted dose fell below 30 Gy-Eq at around 60 mm in depth, which is noted to be a tumor dose necessary for tumor response for BNCT shown by Laramore *et al.* [53] At a depth of 60 mm, the dose derived from thermal neutrons was dominant but declined slightly, then gamma dose contributed 4.4%, whereas fast neutron dose represented 0.7%.

**Table 1:** The absorbed dose composition at each reference point on the beam axis in 200-mm cubic water phantom in BPA-BNCT for brain tumor

Dose Component	RP_surface_			RP_peak_			RP_distal_		
	RBE-weighted dose (Gy-Eq)	(% in tumor)	(% in brain)	RBE-weighted dose (Gy-Eq)	(% in tumor)	(% in brain)	RBE-weighted dose (Gy-Eq)	(% in tumor)	(% in brain)
Tumor total dose	18.80	(100)	–	64.36	(100)	–	30.58	(100)	–
Tumor boron dose	14.87	(79.1)	–	60.83	(94.5)	–	28.57	(93.5)	–
Brain total dose	5.35	–	(100)	9.35	–	(100)	4.74	–	(100)
Brain boron dose	1.42	–	(26.6)	5.82	–	(62.3)	2.73	–	(57.7)
Gamma dose	1.12	(5.9)	(20.9)	1.84	(2.9)	(19.7)	1.36	(4.4)	(28.7)
Nitrogen dose	0.22	(1.2)	(4.1)	0.86	(1.3)	(9.2)	0.41	(1.3)	(8.6)
Hydrogen dose	2.51	(13.3)	(46.8)	0.79	(1.2)	(8.4)	0.22	(0.7)	(4.6)
Others dose^*^	0.09	(0.5)	(1.6)	0.03	(0.1)	(0.4)	0.02	(0.1)	(0.4)

^*^The sum of gamma dose based on reactions occurring between elements and neutrons, except for reactions giving boron, hydrogen and nitrogen doses, which are calculated on SERA based on elemental composition ratios reported by ICRU46 and nuclear data derived from ENDF/B.

Abbreviations: BPA, boronophenylalanine; BNCT, boron neutron capture therapy; RP_exit_, reference point at the center of an exit port on a moderator surface; RP_surface_, reference point at the center of the phantom surface; RP_peak_, reference point at 20-mm depth from RP_surface_ on the center axis in phantom; RP_distal_, reference point at 60-mm depth from RP_surface_ on the center axis in phantom; RBE, relative biological effectiveness.

Based on these beam component ratios for each dose (Table 1), RPs for evaluation were set as the center of an exit port on the moderator surface (RP_exit_), the center of the phantom surface (RP_surface_), and depths of 20 mm and 60 mm from RP_surface_ along the central axis in the phantom (RP_peak_ and RP_distal_, respectively).

Finally, the clinical tolerances of each dose component were calculated as variability of single dose components allowed within 3% of total tissue RBE-weighted dose, as shown in Table 2. On the other hand, when the phantom surface is assumed to be skin, the skin dose consists of the dose components shown in Table 3, and the tolerance levels of the components contributing to each dose component vary. On SERA calculation, there are large errors in the calculation of the surface doses of the anatomy, especially for fast neutrons, with a difference of up to 30–60% [54]. Therefore, the corresponding tolerance level in Table 3 is estimated to be 6.3%, taking into account the worst case of underestimation with respect to the deviation of fast neutron from the simulation. Since this value is smaller than the value in Table 2, 6.3% should be adopted.

**Table 2:** Tolerance of variation for measurement of each dose component

Contribution	Related dose component	Action level at RP_surface_ (%)		Action level at RP_peak_ (%)		Action level at RP_distal_ (%)	
		Tumor	Brain	Tumor	Brain	Tumor	Brain
Thermal neutron	Boron dose Neutron dose	**3.7**	9.8	**3.1**	4.2	**3.2**	4.5
Fast neutron	Hydrogen dose	22.6	**6.4**	(100)	**35.7**	(100)	**65.2**
Gamma	Gamma	50.8	**14.4**	(100)	**15.2**	68.2	**10.5**

Calculated as variability of single dose components allowed within 3% of total tissue RBE-weighted dose.

The notation ‘(100)’ indicated that the value exceeded 100. The bold numbers indicate the definitive values adopted as clinical tolerance levels for the relevant dose component at each RP.

Abbreviations: RBE, relative biological effectiveness; RP_surface_, reference point at the center of the phantom surface; RP_peak_, reference point at 20-mm depth from RP_surface_ on the center axis in phantom; RP_distal_, reference point at 60-mm depth from RP_surface_ on the center axis in phantom.

**Table 3:** Skin dose composition and the tolerance of variation for measurement at RP_surface_

Dose Component (Gy-Eq)	RBE-weighted dose (Gy-Eq)	(%)	Contribution	Tolerance level (%)
Skin total dose	6.59	(100)	–	–
Boron dose	2.65	(40.3)	Thermal neutron	7.4
Nitrogen dose	0.34	(5.2)		
Hydrogen dose	2.39	(36.3)	Fast neutron	8.3
Gamma dose	1.12	(16.9)	Gamma	17.7

Tolerance levels were calculated as variability of single dose components allowed within 3% of total tissue RBE-weighted dose.

Abbreviations: RP_surface_, reference point at the center of the phantom surface; RBE, relative biological effectiveness.

### Established accelerator-based BNCT QA program

The irradiation field of BNCT contains neutrons with a broad energy spectrum and gamma. Therefore, strictly, in order to keep dosimetric error within 3%, measurements for dosimetric QA must be considered for each of the four typical radiation dose, such as thermal/epithermal neutrons, fast neutrons, and gamma. However, since each dose component has a very different spatial distribution and also has a different RBE, it is rather less meaningful to guarantee uniformly 3% accuracy for all component physical doses at all RPs. It is more important to secure required accuracy for each dose component based on contribution to the total RBE-weighted dose at each RP instead of guaranteeing with RBE-non-weighted physical dose. Based on these concepts, with regards to these contributions at each RP, daily, monthly, and annual QA items were arranged according to AAPM TG-142 and TG-198 in Table 4 and Supplementary Table A2–4.

**Table 4:** The detailed procedures of cyclotron-based BNCT’s quality assurance optimized for the hospital use

**Dosimetric QA items**	**Procedures and tolerances** ^*^ **for each beam component**		
	***Thermal and epithermal neutron***	***Fast neutron***	***Gamma***
**Daily QA**			
*Output constancy*	Reaction rate of a gold foil after irradiation with 0.3 C at RP_exit_ Tolerance: ±2.0%	—	—
**Complementary weekly QA**			
*Output constancy*	Reaction rate of gold foils after irradiation with 0.3 C at RP_surface_, RP_peak_, RP_distal_ Tolerance: ±2.0% at RP_surface_, and RP_peak_; ±2.5% at RP_distal_	—	Absorbed dose calculated from TLD after irradiation with 0.6 C at RP_surface_, RP_peak_, RP_distal_ Tolerance: ±7.0% [±14.4% at RP_surface_, ±15.2% at RP_peak_, ±10.5% at RP_distal_]
**Monthly QA**			
*Output constancy with charge amount monitor calibration*	*If needed.* *Same as monthly QA with different tolerance level.*	—	*If needed.* *Same as monthly QA with different tolerance level.*
*Beam profile constancy*	Reaction rate of a gold wire after irradiation with 0.5 C at the points with at least 20-mm intervals on the center axis, including RP_surface_, RP_peak_, and RP_distal_ Tolerance: ±2.0% from baseline	—	Absorbed dose calculated from TLD after irradiation with 0.6 C at the points with at least 20-mm intervals on the center axis, including RP_surface_, RP_peak_, and RP_distal_ Tolerance: ±7.0% from baseline
**Annual QA**			
*Output constancy (of fast neutron)*	—	Reaction rate of indium foils and resulting acquired absorbed dose rate derived from fast neutron after irradiation with 1.8 C at RP_surface_ Tolerance: 10.5%	—
*Symmetry change from baseline*	Reaction rate of gold wires after irradiation with 2.8 C at the points with appropriate interval and range on both off-axis principal axes x and y at depth of RP_peak_ and RP_distal_ Tolerance: ±3.0% from baseline	—	Absorbed dose calculated from TLD after irradiation with 0.6 C at the points with appropriate interval and range on both off-axis principal axes x and y at depth of RP_peak_ and RP_distal_ Tolerance: ±7.0% from baseline
*Beam quality*	Reaction rate of a gold wire with and without cadmium cover, and resulting acquired thermal neutron flux after irradiation with 1.2 C at the points with at least 10-mm intervals on the center axis, including RP_surface_, RP_peak_, and RP_distal_. The percentage depth values (PDV) compared to RP_peak_, are evaluated. Tolerance: ±3.0% from baseline [±3.7% at RP_surface_; ±3.1% at RP_peak_; ±3.2% at RP_distal_]	—	Absorbed dose calculated from TLD after irradiation with 0.6 C at the points with at least 10-mm intervals on the center axis, including RP_surface_, RP_peak_, and RP_distal_ Tolerance: ±7.0% from baseline [±14.4% at RP_surface_; ±15.2% at RP_peak_; ±10.5% at RP_distal_]
*The linearity between charged amount monitor and output constancy*	Reaction rate of gold foils after irradiation with at least 5 different charge amount around 0.3–4.2 C at RP_peak_ Tolerance: ±3.0% from a regression line	—	—

^*^Square brackets represent the values derived from clinical tolerances. All other listed tolerance values were determined empirically based on ±2SD from 5–10 consecutive measurements.

Abbreviations: BNCT, boron neutron capture therapy; QA, quality assurance; RP_exit_, reference point at the center of an exit port on a moderator surface; RP_surface_, reference point at the center of the phantom surface; RP_peak_, reference point at 20-mm depth from RP_surface_ on the center axis in phantom; RP_distal_, reference point at 60-mm depth from RP_surface_ on the center axis in phantom; TLD, thermoluminescence dosimeter.

It is necessary to adopt measurement method with guaranteed accuracy and stability for dosimetric QA. Therefore, it was most appropriate to use classical activation method for evaluation of neutron and use of real-time neutron detectors for which stability and durability have not yet been confirmed was omitted.

For assessment of neutron flux with a set of gold activation with and without a cadmium cover, and gamma using TLD, on the condition that measurement uncertainty (*k* = 2) calculated from the repeated measurement values was within the limits of the clinical tolerances, the institutional tolerance level for each measurement were determined based on the value of ±2 SD or reasonable variation range based on permissible empirical errors of measurement. In evaluations using activation reaction rates based on gold or indium in which no neutron flux is derived, since there is no linear relationship between neutron energy and reaction cross section, and the reaction rate of gold or indium based on measurement is not a quantitative indicator of neutrons. Therefore, for these measurements, the institutional QA tolerance level was determined based on the measurement uncertainty (*k* = 2), without considering the clinical tolerance derived from the RBE dose.

### Output constancy

**Fig. 3:**
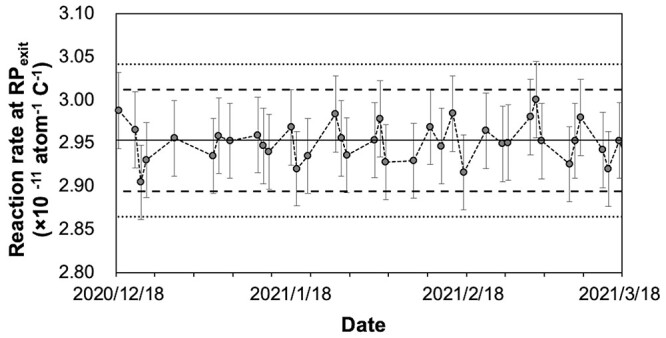
Daily variation of the gold reaction rate. Daily variation of the reaction rate measured with the activation of the gold foils at RP_exit_. Error bars indicate the measurement uncertainty (k = 2) calculated from reference data based on 10-times measurements. The solid line depicted the average of the reference data. The institutional tolerance level of the measurements for daily variation was determined as ±2.0% from reference value, represented as broken lines. The dotted line depicted ± 3.0% as a reference. Abbreviations: RP, reference point.

**Fig. 4:**
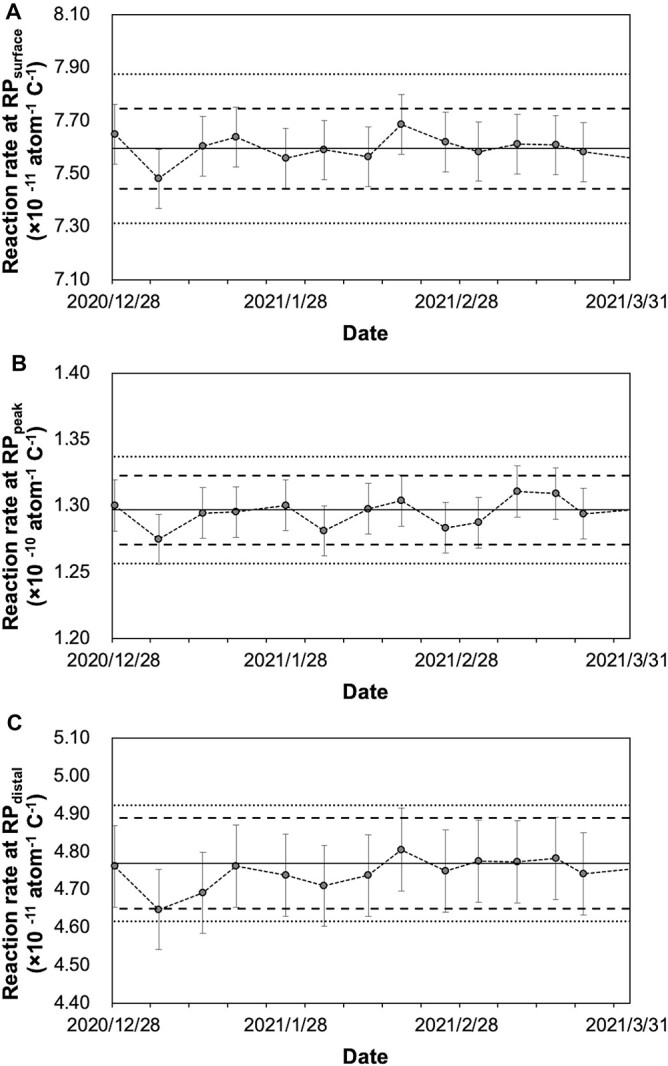
Weekly variation of the gold reaction rate. Weekly variation of the reaction rate measured with the activation of the gold wires at RP_surface_ (A), RP_peak_ (B) and RP_distal_ (C). The measurement uncertainty (k = 2) calculated from reference data (*n* = 10) for weekly variation at RP_surface_, RP_peak_ and RP_distal_ were 1.5%, 1.7% and 2.3%, respectively, represented as error bars. The institutional tolerance levels of the measurements at RP_surface_, RP_peak_ and RP_distal_ for weekly variation were determined as ±2.0%, ±2.0% and ± 2.5% from reference value, represented as broken lines. The dotted lines depicted ± 3.7%, ± 3.1%, and ± 3.2% as references with regards to the values of clinical tolerances for thermal neutron derived from the permitted variation of total RBE-weighted dose. Abbreviations: RP, reference point.

**Fig. 5:**
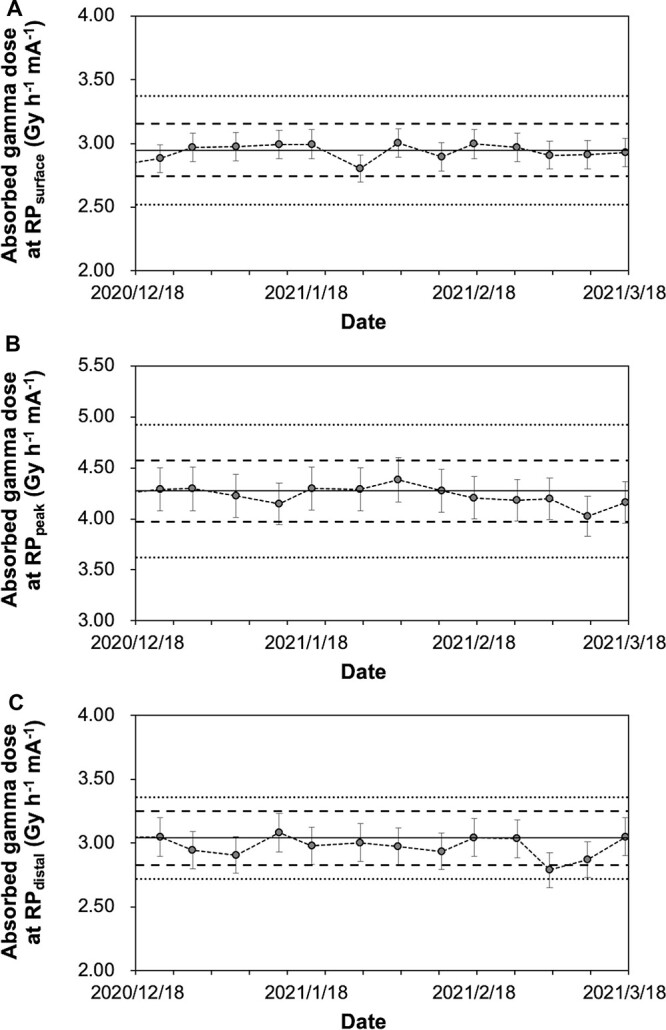
Weekly variation of the gamma dose. Weekly variation of the gamma dose measured with TLD at RP_surface_ (A), RP_peak_ (B) and RP_distal_ (C). The measurement uncertainty (k = 2) calculated from reference data (*n* = 13) for weekly variation at RP_surface_, RP_peak_ and RP_distal_ were 3.8%, 4.9% and 4%, respectively, represented as error bars. The institutional tolerance levels of the measurements at RP_surface_, RP_peak_ and RP_distal_ for weekly variation were determined as ±7.0%, ±7.0% and ± 7.0% from reference value, represented as broken lines. The clinical tolerances from the permitted variation of total RBE-weighted dose were ± 14.4%, ±15.2% and ± 10.5% from reference value, represented as dotted lines. Abbreviations: TLD, thermoluminescence dosimeter; RP, reference point; RBE, relative biological effectiveness factor.

For daily QA, as a pre-start inspection, normal operation of a proton beam current monitor of the accelerator is confirmed, and the electrical stability and output constancy of the device are evaluated. Adopting gold activation method, which is considered to be the most reliable, in order to complete the measurement within 30 min, irradiation with a constant charge of 0.3 C, which is equivalent to 5 min irradiation with a proton beam current of 1 mA, to a gold foil positioned at RP_exit_ without using phantom was performed. Since the epithermal neutron flux near a beam aperture is 1.2 × 10^9^ cm^−2^ s^−1^, which is significantly larger than the thermal neutron flux of 5.0 × 10^6^ cm^−2^ s^−1^ [29], this measurement reflects mainly epithermal neutron which will be converted to thermal neutron in the human body or phantom. And the reaction rate calculated from a net count of the activity of gold foils by HP-Ge was evaluated as alternatives to thermal/epithermal neutron flux (Fig. 3). In our preliminary experiment, the lowest charge level at which the measurement uncertainty reduction almost reached a plateau at RP_exit_ was 0.3 C, and this charge level was adopted. A pre-check of the proton current monitor being within 1% deviation prevented uncertainty of the delivered proton charge from propagating to the evaluation. As a result of the examination of measurement dispersion in which the relationship between the net count number on HP-Ge, the counting time and standard deviation of reaction rates were compared with up to 1 × 10^5^ net counts, and the required daily net count number was decided as 5 × 10^4^, with which the counting time could be kept within 5 min and total evaluation time kept within 15 min. The evaluation of gamma using TLD was omitted because it was assumed to require at least 90 min of additional time. In addition, the TLD measurements of fluorescence intensity with a short time were attempted preliminarily, but the accuracy of the measurements was not stable during the period in which fluorescence intensity is exponentially fading. To compensate for the shortage in daily output constancy testing, as a weekly dosimetric QA for output constancy, the reaction rate of gold foils immediately after irradiation with 0.3 C and the gamma dose calculated from TLD-indicating dose 1 hour after irradiation with 0.6 C at RP_surface_, RP_peak_ and RP_distal_ were examined and constancy of these values was evaluated. The variation of reaction rates of gold foils and indicated values of TLD were shown in Fig. 4 and Fig. 5. When the neutron energy spectrum is deviated toward lower energy side, the flux of thermal and epithermal neutrons that can reach deep into the tissues decreases, and conversely, when it is deviated toward higher side, they increase. Therefore, the change in the neutron energy spectrum may be reflected in the change in the measured neutron beam profile. When the constancy of the reaction rates at RP_surface_, PR_peak_, and PR_distal_ was maintained, it was assumed that the constancy of the neutron energy spectrum was ensured at a certain level.

As a monthly output constancy QA, the proton beam current monitor is validated using external equipment secured with traceability, and calibrated if the values are over a range of tolerance level. If calibration is required, daily and weekly output constancy QA is carried out after calibration to confirm constancy in comparison with reference values.

As an annual QA, additional constancy of the RBE-weighted dose derived from fast neutron was evaluated by the reaction rate of indium. The unnecessary reactions of ^115^In(n,γ)^116m^In, was difficult to be prevented completely by the indium foil covered with 0.7-mm cadmium. As a result, when the photon counting was started immediately after irradiation by an HP-Ge, the photon peak of ^115m^In could not be observed due to the scattering effect of the spectra from the high energy side of ^116m^In. As an optimized measurement condition, at the RP_surface_ point, which is assumed to have the highest fast neutron flux (Fig. 2), continuous photon counting were carried out for 8 h after a cooling time of 13 h, following 30 min irradiation, and finally sufficient photon counts were acquired with counting uncertainty with *k* = 2 of less than 5%. However, the dispersion of several consecutive measurements was large with a 2SD of 10.5%, and the measurement uncertainty based on Type A evaluation of the finally acquired reaction rate with *k* = 2 was still very large with 5.3% (Table 4). For this reason, it was difficult to determine whether output constancy of fast neutron was secured within a clinical tolerance, then this value was treated as a reference, although this uncertainty was still less than 15–20% articulated in IAEA-TECDOC-1223 [55]. In any case, measurement precision can become poor even after optimizing the measurement condition at best. Furthermore, the threshold energy of the indium foil is approximately 1.2 MeV, and the energy region from 10 keV to 1.2 MeV is not evaluated. Therefore, the dosimetric accuracy of the fast neutron should be evaluated by considering whether there is any variation in the reaction rate of gold foils in each RP. The measurement of fast neutrons was omitted from the daily, weekly, and monthly QA because the poor results based on the low measurement precision were not commensurate with such a high workload (Supplementary Table A2–4). Development of a high-precision evaluation method for fast neutrons should be considered a future issue.

### Beam profile constancy and beam quality QA

The constancy of neutron beam property was planned to be confirmed based on the gold activation reaction rate for monthly beam profile constancy QA and the thermal neutron flux for annual beam quality QA to confirm the constancy of the beam properties at each RPs. As well, the gamma beam property was planned by monthly and annual QA. As a monthly QA, evaluation of the beam profile was planned using depth distributions for the reaction rate of fragments cut from a single gold wire for each depth and gamma doses acquired from TLD indicating dose on the beam axis. Evaluation based on the activities of Indium foils reflecting fast neutrons was not adopted because of extremely poor contribution to the total weighted dose in almost all depth except for RP_surface_ in spite of time-consuming procedure on operation flow. The distribution curves of the gold wire reaction rates immediately after irradiation with 0.5 C for gold wires (due to multipoint simultaneous measurements in contrast to 0.3 C in the daily output constancy QA) and absorbed gamma dose 1 hour after irradiation with 0.6 C for TLDs at the points with at least 20-mm intervals including RP_peak_ and RP_distal_ on the central axis were evaluated.

As an annual beam quality QA, evaluation of the beam quality profile by thermal neutron flux at points with 10-mm intervals as well as the gamma dose at points with 10-mm intervals on the central axis was adopted. As in the evaluation of the beam profile, 100-mm gold wires were irradiated on the central axis in the water phantom without and with a cadmium cover, located based on the laser in the irradiation room. The irradiated gold wires were fragmented from the proximal end and measured to derive the thermal neutron flux. Since the thermal neutron flux is calculated based on the mass of gold, the measurement uncertainty of the precision electronic balance used for the measurements is compounded into the final flux uncertainty. When the gold wire is measured in fragments of 5 mm each, the measurement uncertainty component for the electronic balance (*k* = 2) in a single measurement was 2.2%. For evaluation of thermal neutron flux, two independent measurements with and without cadmium cover would give twice this uncertainty component, resulting in a synthetic uncertainty of 3.1%, even if the other all uncertainty components are excluded. However, by repeated mass measurements and minimized deviation of cut position of gold wire as well as ensuring adequate counting of Hp-Ge detector, the overall uncertainty based on actual measurements was controlled within 3.0% at all points. The presence or absence of HP-Ge detection efficiency deterioration was evaluated based on measurements with standard sources (Ba-133, 2088-49-1, Eckert and Ziegler Isotope Products, CA; Co-60, 2070–85, Eckert and Ziegler Isotope Products; Cs-137, 2070–86, Eckert and Ziegler Isotope Products) at least once a week. The values of the adopted detection efficiencies were reviewed every three to six months as instrument settings were updated or after earthquakes. In each of these periods, the variation in the measured values of detection efficiency was kept within ±0.50% as ±2SD. The measurement uncertainty of thermal neutron flux was greatly improved from the value of 5–7% documented by the IAEA [55]. Since the values were all lower than the clinical tolerances as a reference at each point, ± 3.0% from baseline was reasonable as the institutional tolerance. Evaluation of percentage depth values compared to RP_peak_, equivalent to the percentage depth dose for linac, were considered. Occasionally, a polynomial approximation is performed for the beam shape on the beam axis, and the deviation of each RP from the reference is recorded as the difference. However, since measurement errors at other points affect the validity of the approximation, the validity of the obtained deviation should be judged taking into account the deviation of each measurement point from the reference and other recent results of QA measurements. Beam profile as a baseline obtained by past measurements is shown in Supplementary Fig. A and B.

### Off-axis distribution change from baseline

As an annual QA, the beam symmetry profile of gold reaction rates and gamma dose, and the resulting ratio of values at each symmetric position, equivalent to the off-axis ratio (OAR), were planned as an annual QA. The beam profiles as baseline obtained from past measurements are shown in Supplementary Fig. C. Because of the lack of flatness in the off-axis distribution, the flatness test was not applied.

### Proton beam current monitor calibration and proton charge value linearity on output constancy

Validation of a proton current monitor DCCT (DC current transformer) is very important because the accuracy of activation method depends on that of proton current monitor. The stability of the proton current monitor was very robust and it has never required calibration in its 5 year use in our facility. Relying on this robustness, we have recognized that it is sufficient to ensure stability in daily and monthly output constancy QA which equal to performing at least once a week. Evaluation of linearity between the proton beam charge and the reaction rate of gold wires was considered as an annual QA. The profiles as baseline obtained from past measurements are shown in Supplementary Fig. D. It was confirmed that the deviation of each data point from the regression line were within the range of these tolerance level within ±3%.

### Handling of measured values and tolerance levels

If the measured value exceeded the tolerance level, it was assumed that the measurement would be repeated, the metal sample would be re-weighed, and the logs of the irradiation device and HP-Ge detector would be checked, and photographs and other records would be checked to confirm whether there was any setting error. If no problem was found in the above contents affecting measurement error, re-irradiation and re-measurements would be performed and it would be checked whether there is a similar deviation trend. And if a trend is found, it would be planned to conduct a detailed investigation of the irradiation device under the manufacturer’s survey.

## DISCUSSION

As a method of dosimetric evaluation which has been carried out in nuclear reactors so far, the technique of the neutron activation analysis for neutrons has been established, and the emphasis has been given to assure the desired epithermal neutron flux with more than 1 × 10^9^ cm^−2^ s^−1^ as described in the International Atomic Energy Agency (IAEA) tecdoc [56]. However, since the RBE-weighted dose administered to tissues depends on each component of the mixed radiation in the irradiated field, in order to ensure the entire RBE-weighted dose, the ratio of each RBE-weighted dose component at each reference point must be considered.

There are currently insufficient reports on QA methods for BNCT. Rassow *et al.* reported on QA matters for BNCT to be implemented to maintain quality equivalent to the QA for electron accelerators [57]. Although an outline of QC/QA actually adopted in a nuclear reactor was described, this cannot be considered sufficient when compared with the QA requirements of a linear accelerator [32]. The QA requirements presented here represent the first concrete description of QA that enables use of an accelerator-based BNCT system in daily clinical practice, and the QA requirements to be considered are adequately incorporated in comparison with the linear accelerator. Therefore, this study provided a basis for establishing a QA methodology for the accelerator BNCT system considering the current technical limitations and the management in medical institutions. We finally concluded that action level in each QA measurement should be the variation (%) equivalent to 3% deviation of total RBE-weighted dose for each dose component, and the tolerance level for each measurement would be considered with ±2 SD or reasonable variation based on empirical measurement results. For some measurements, we determined the tolerance levels based on 2SD, which indicates the dispersion of the measurement, rather than on 2SD of means commonly adopted as the measurement uncertainty. This is because the precision of each measurement method is not high enough, and using the 2SD of means as a measurement uncertainty increases the risk of underestimating the uncertainty. In fact, when the 2SD of means have been adopted as the tolerance level for thermal neutron flux, gold reaction rate, and TLD measurement, the measured values frequently deviated from the tolerance level, and it was thought that the uncertainty was underestimated. Therefore, we considered it appropriate to adopt the 2SD. These tolerance levels are comparatively larger compared to those adopted in dosimetric QA for the linear accelerator, and therefore the results of the measurements must be carefully assessed for quality. The relatively lower quality of this dose assurance should be considered a specific characteristic of BNCT that must be accepted as a difference from that of conventional radiotherapy. To further improve the quality of dose assurance, if an easier-to-use neutron detector with sufficient measurement accuracy is developed, its installation should be considered. The methodology for QA in this report was provided by an instrument that combines a beryllium target with a 30-MeV cyclotron. When this methodology is applied to BNCT using neutron sources with different specifications, it is necessary to consider that the contamination ratios of the radiation dose components are somewhat different depending on each instrument configuration. That is, it is necessary to provide a clinical tolerance for each instrument and then determine the tolerance value of each component dose for QA procedures.

Since this QA concept was devised prior to the JG002 phase II clinical trial of accelerator-based BNCT for malignant glioma, the clinical tolerances were given primarily for brain tumors. When mucosal doses are taken into account in consideration with BNCT for head and neck cancer, the boron concentration ratio and CBE factor for mucosa membrane are higher than normal brain tissue, relatively, although not as much as for brain tumors. And, the dose component ratio is, briefly, somewhere between those of normal brain tissue and brain tumors considered in this study. Therefore, clinical tolerance of the mucosa in BNCT for a head and neck region has already been achieved as long as clinical tolerance in normal brain tissue is ensured. At present, it is impossible to know the true values of tissue boron concentration and CBE factor, and treatment is performed by giving these values as assumed constant values. This inaccuracy must be recognized in post-treatment evaluation of clinical efficacy and adverse events by radiation oncologists specializing in BNCT. The situation is not necessarily different to that in conventional radiotherapy, where the presence or absence of hypoxic tumor fractions is not taken into account in dose calculation due to unknown nature, even though the RBE of the tumor are strongly affected by the oxygen conditions. Normal tissues are likely to be more constant in characteristics, such as the concentration of boron agents and the CBE factor, than tumor tissues which have different properties along with each patient. In this study, clinical tolerances of each QA procedures were decided based on brain tumor and normal brain tissue. However, depending on the specific conditions of irradiation, clinical tolerance may need to be considered for other organs with more strict dose requirements. These weighted doses are reported to be overestimated, especially on the high dose side, rather than iso-effective dose, which is more indicative of actual biological equivalent doses. If the iso-effective doses are taken into account, a lower contribution of boron doses on the high dose side is expected, and the tolerance level of thermal neutrons as a weighted dose is more mitigated. Therefore, it is unlikely to cause significant practical problems if the constancy of the equipment is guaranteed at the current tolerance levels based on the RBE/CBE-weighted dose. However, since the iso-effective dose is based on cell surviving curve data, there is no way to know what the actual tissue or tumor surviving curve of the patient looks like when introducing this concept in clinical practice, and more complex speculation is required.

For measurement of dosimetry derived from thermal and fast neutron, it is common to use the metal activation analysis as the most reliable measurement as long as the workload is more optimized from an operational point of view. Uncertainties in the linearity of the gold wire, fragmentation of the gold wire, and mass measurement of the fragmented gold wire, all of which are included in procedures of thermal and epithermal neutron measurements, are reflected in the final measurement uncertainty. If the uncertainty due to the measurement position deviation is difficult to reduce, the use of a solid phantom such as PMMA can be attempted. Although real-time dosimeters have been developed and studied, such as Si-diode-based detectors, ionization chamber type dosimeters, and scintillator-based detectors [39–42, 57–64], none of these has been reported as simplified options for preparation and measurement optimized for daily use. Also, the risk of low constancy of the detector itself, such as the fall of sensitivity due to denaturation of neutron detector still remain. Therefore, after all QA procedures using metal foil activation method is needed every time for use of these real-time detectors as a daily cross validation [40–42, 57, 63]. In this study, the daily dosimetric QA procedure using the activation method was optimized to be completed within 30 min, including the time for preparation. Also in the newly developed real-time detectors, preparation should preferably be conveniently completed with a simplified daily procedure, the same as in the activation analysis. On the other hand, in real-time confirmation of the constancy of the beam under irradiation, the real-time detector is considered to play a complementary role in charge of the proton beam current monitor of the accelerator. Using a real-time detector in the evaluation of the annual beam profile and symmetry change, the geometrical continuity of these profiles on the beam axis and off axis can be determined.

For measurement of fast neutron, it was difficult to determine whether output constancy of fast neutron has been ensured within a clinical tolerance of 6.4% from the measured values, although total measurement procedures take over 21 h for once (Supplementary Table A4). Therefore, the fast neutron output constancy was evaluated only for annual QA in consideration of the time and effort required for the measurement. Since a neutron energy spectrum will change along with the change of thermal and epithermal neutron fluxes, it was considered to be practical to assume that if there was no change in the profile of the activation rate of gold at each measurement point derived from weekly and monthly QA for thermal and epithermal neutrons, there would be no significant change in fast neutrons. Even the measurement of a single indium foil imposes a load on the irradiation system (the carbon foil stripper in the accelerator needs to be replaced every 3–5 hours of cumulative beam-on time), and occupies the HP-Ge system for a long time, preventing other QA measurements from being performed and ultimately limiting the patient treatment slots. Therefore, it was difficult to incorporate the fast neutron constancy test into the daily, weekly and monthly QA procedures. In order to solve this problem, the installation of multiple expensive HP-Ge systems should be considered if the facility allows it. Or the combination of the moderated fast neutrons into thermal neutrons by using combination of moderators, such as Bonner spheres, and the gold activation method or the simplified measurement method using real-time detectors need to be considered. However, there is no report yet on how much the counting of moderated fast neutrons with low flux can be ensured by the gold activation method or the real-time detector, and to what extent the measurement uncertainty can be reduced. This is an issue to be discussed in the future. Especially for the skin dose directly affected by fast neutrons, when simulated on the surface of the cubic phantom under the same conditions of this study and with the skin CBE factor of 2.5, 36% (2.4 Gy-Eq) of the total skin RBE-weighted dose (6.6 Gy-Eq) was a fast neutron-dependent dose. In a previous report on BNCT for melanoma using a nuclear reactor, 18 Gy-Eq was administered as a skin maximum dose. For BNCT with such a high skin dose, a more frequent output constancy evaluation for fast neutron may be required, despite the large measurement uncertainty [65].

Under our QA methodology, the gamma dose measured by TLD include dose originating from the ^1^H(n,γ)^2^H reaction, and are measured to check whether constancy is guaranteed in the gamma dose distribution in the tissue. Since the fundamental concept is to ensure that there is no change in the composition of the RBE dose in the tissue, we consider it acceptable to measure and evaluate the gamma dose including that from ^1^H(n,γ)^2^H reaction using a phantom. Deviating from this concept, if it is possible to evaluate the variation in system-derived gamma dose only, excluding the dose from ^1^H(n,γ)^2^H reaction, of course that would be ideal. However, then we must consider whether it is possible to simply evaluate the gamma dose deviation with an adequate precision. For example, according to actual measurements for our system, the gamma dose without phantom at a beam aperture is measured as 30% of that with the water phantom at the RP_surface_. Consequently, if we adopt the same measurement conditions as measured with the phantom, the uncertainty in the measurement of gamma dose without the phantom will simply exceed 20%. Therefore, it is difficult to determine whether or not constancy is ensured based on measurement without the phantom. On the other hand, if the device-derived gamma dose deviation from the standard value exceeds over 20%, it can be detected also in the measurement using the phantom.

For gamma dose measurement, it is necessary to use a detector insensitive to mixed neutrons. Since thermal and epithermal neutrons are absorbed by lithium fluoride with sufficient thickness more than several mm, it is considered reasonable to use a gamma ray dosimeter such as TLD elements covered with this. However, due to the influence of the thickness of lithium fluoride, the detector size become large so that measurement points are limited to the points with large distance with each other, such as with interval of over 20 mm. Therefore, in this study, use of a thin TLD element encapsulated in a capillary tube made of quarts in place of lithium fluoride cover was indispensable. Although TLD-700, composed of ^7^LiF:Mg,Ti containing 99.99% of Li 7 and 0.003% of Li 6, has many use records in nuclear reactors as a commercially available element, the response to thermal neutrons still has contributed to 83% of the signal and it has been founded that a correction factor of 0.17 is needed to indicate the gamma dose [66]. The large proportional uncertainty of gamma dose for TLD-700 ranged from 10% to 40% has been reported [67]. Although Tsai *et al.* reported that the TLD-400 chip composed of CaF2:Mn has negligible sensitivity to thermal neutrons in irradiation experiment with THOR, suggesting its usefulness in gamma dose measurement [66], further investigation is required on the possibility of use of TLD-400 in dosimetric QA.

In conclusion, we have systematically established a methodology for dosimetric QA of commercial BNCT systems using an accelerator. These results provide a feasible QA method that can be clinically applied with a certain dosimetric validity for the mixed irradiation field of BNCT.

## CONFLICT OF INTEREST

H.T. reports personal research funding from Sumitomo Heavy Industries, Ltd.. The other authors have nothing to declare.

## FUNDING

Supported by Fukushima prefectural subsidy for development and testing of global cutting-edge medical devices.

## Supplementary Material

SupplementaryFigures_20220119_rrac030

SupplemtaryTables_rrac030

SupplementaryData_flux_calculation_rrac030

## Notes

(Received Month Day, 2022; revised Month Day, 2022; Editorial Decision Month Day, 2022)
