# Supplementary material for: Determining a methodology of dosimetric quality assurance for commercially available accelerator-based boron neutron capture therapy system
Source: J Radiat Res. 2022 Jun 20;63(4):620–35. doi: 10.1093/jrr/rrac030 (PMC9303606; doi:10.1093/jrr/rrac030)

Figure A. Distribution curves of reaction rate of gold wires and epithermal neutron flux on the central beam axis

(a) Distribution curves for reaction rate of gold wires (solid line) and a gold wires coverd with Cd (broken line) were depicted. (b) From these activation rates, distribution of thermal neutron flux was derived. Each point and error bar represent the mean and ±2SD for 6 measurements.

(a)


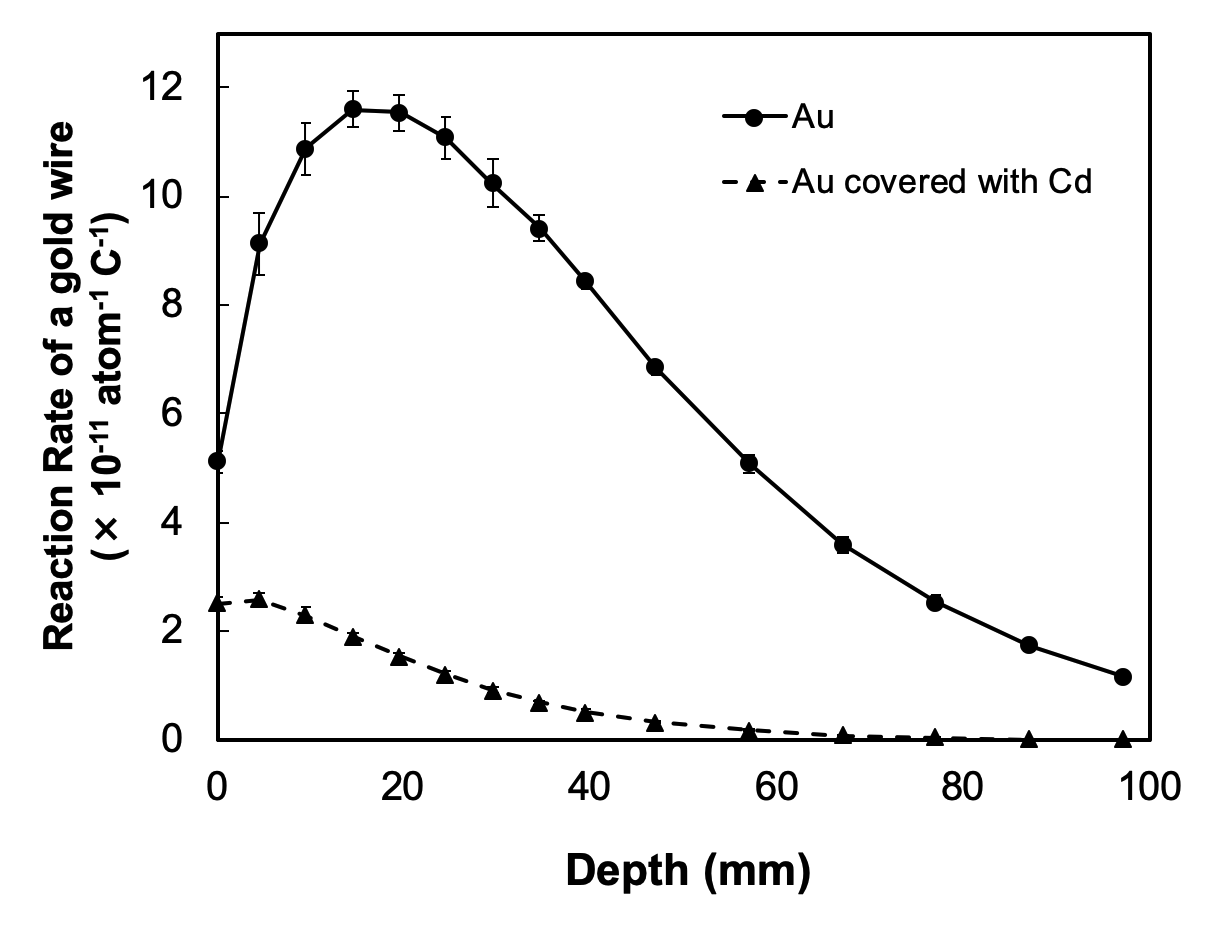


(b)


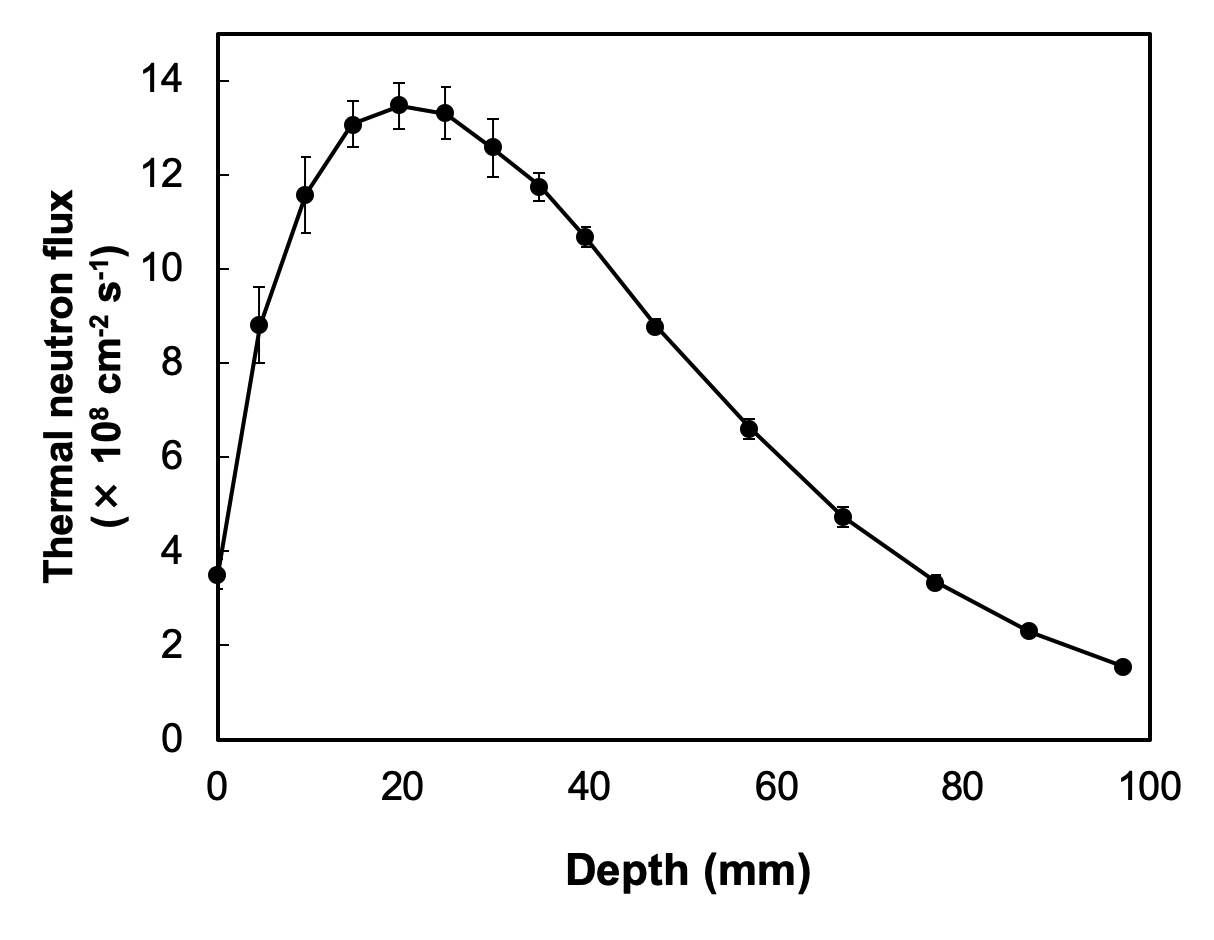


Figure B. Distribution curve of gamma dose on the central beam axis.

Distribution curves of gamma dose was depicted.


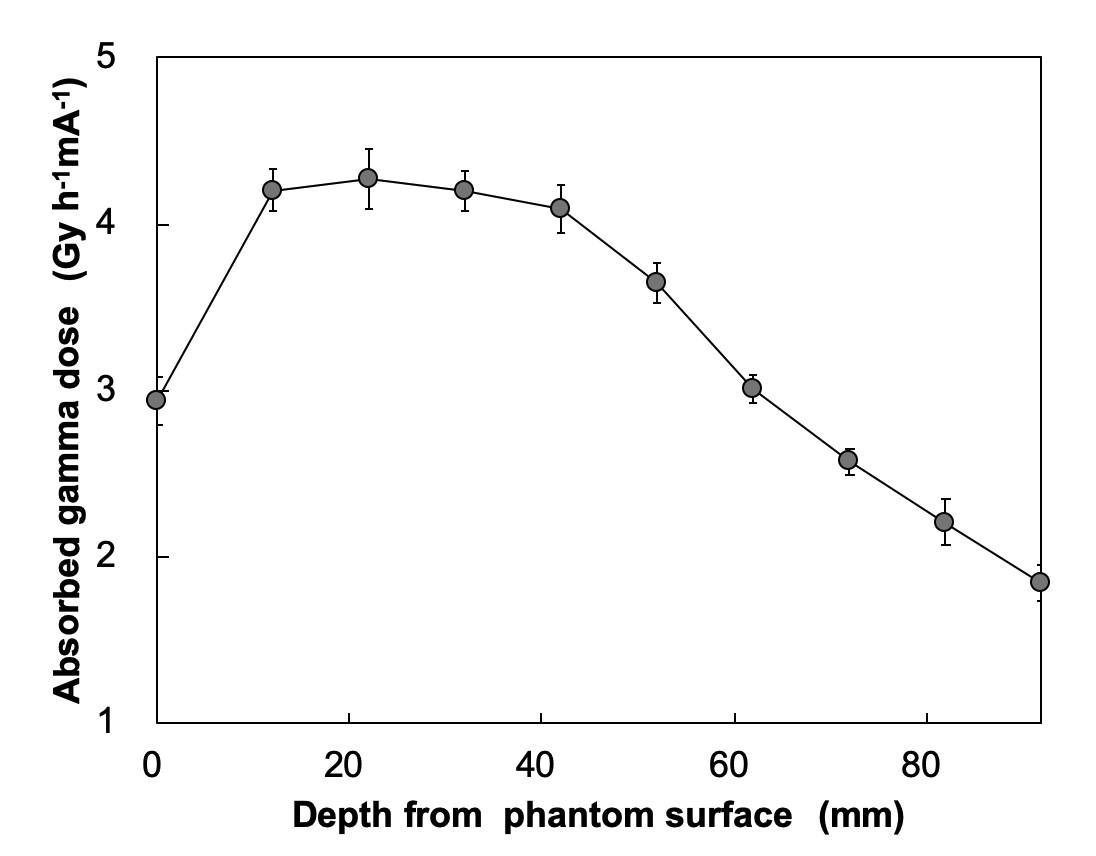


Figure C. Off-axis beam symmetry profile for reaction rate of gold wires

The off-axis beam symmetry profile for the reaction rate of gold wires at RP_peak_ and RP_distal_ are depicted.

Abbreviation: RP, reference point.


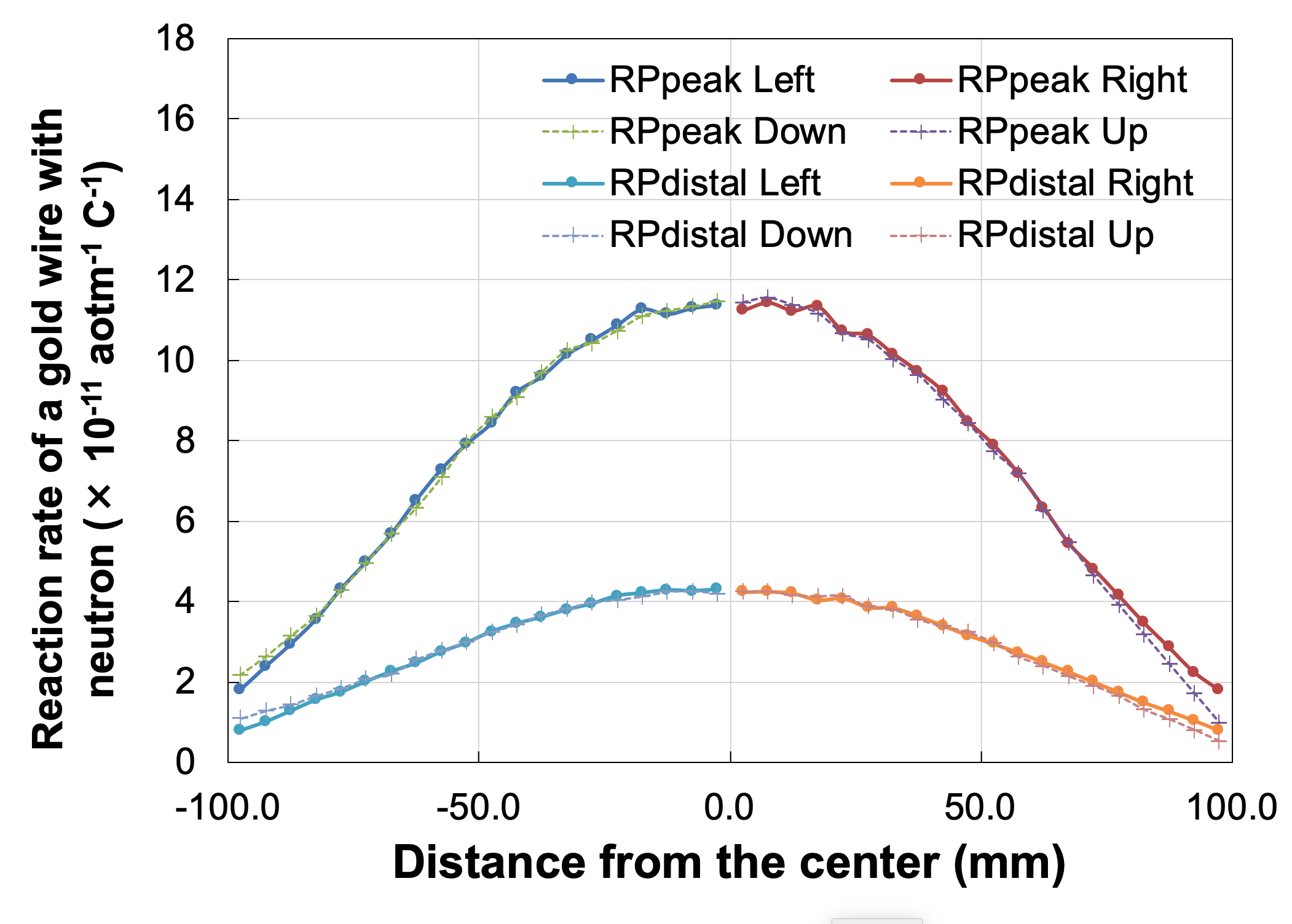


Figure D. Linearity of relationship between the proton beam current and reaction rate of gold wires.

Abbreviation: RP, reference point.


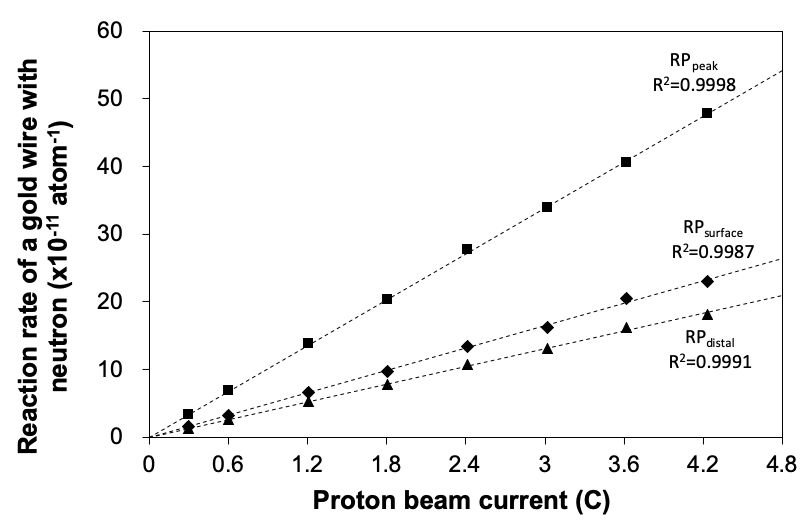

Supplement: SupplementaryFigures_20220119_rrac030 [file supplementaryfigures_20220119_rrac030.docx]
