# Supplementary material for: Determining a methodology of dosimetric quality assurance for commercially available accelerator-based boron neutron capture therapy system
Source: J Radiat Res. 2022 Jun 20;63(4):620–35. doi: 10.1093/jrr/rrac030 (PMC9303606; doi:10.1093/jrr/rrac030)
Supplement: SupplemtaryTables_rrac030 [file supplemtarytables_rrac030.docx]

Table A1. Parameters used in simulation

| Tissue type | Boron CBE | Nitrogen RBE | Hydrogen RBE | T/B | Nitrogen density | Hydrogen density | Carbon density | Oxygen density |
| --- | --- | --- | --- | --- | --- | --- | --- | --- |
| Brain tumor | 4.0 | 2.9 | 2.4 | 3.5 | 2.2 | 10.7 | 14.5 | 71.2 |
| Normal brain | 1.34 | 2.9 | 2.4 | 1.0 | 2.2 | 10.7 | 14.5 | 71.2 |
| Skin | 2.5 | 2.9 | 2.4 | 1.0 | 4.2 | 10.0 | 20.4 | 64.5 |

The other RBEs were fixed as 1.0 for all tissue types. Blood ^10^B concentration was fixed as 25 ppm.

Abbreviations: CBE, compound biological effectiveness; RBE, relative biological effectiveness; T/B, tissue [^10^B]-to-blood [^10^B] ratio.

Table A2. Comparison of dosimetric daily, and weekly QA procedures in accelerator-BNCT with those in AAPM TG-142 and TG-198 report

| **Dosimetric QA procedures in AAPM TG-142 and TG-198 report^*^** | | |  | **Dosimetric QA procedures for accelerator-BNCT (*this study*)** | | |
| --- | --- | --- | --- | --- | --- | --- |
| **Procedure** | **Tolerance** | **Time required (range)** |  | **Procedure** | **Tolerance^**^** | **Time required**^***^ |
| ***Daily QA*** |  |  |  | ***Daily QA*** |  |  |
| Photon and Electron Output Constancy | ±3% | 10–20 min |  | Thermal/Epithermal Neutron Output Constancy (Au RR) | ±2.0% at RP_exit_ | 15 min |
|  |  |  |  |  |  |  |
|  |  |  |  | ***Weekly QA*** |  |  |
|  |  |  |  | Thermal/Epithermal Neutron Output Constancy (Au RR) | ±2.0% at RP_surface_ and RP_peak_; ±2.5% at RP_distal_ | 50 min |
|  |  |  |  | Gamma Output Constancy | ±7.0% at RP_surface_, RP_peak_, and RP_distal_  [±14.4% at RP_surface_; ±15.2% at RP_peak_; ±10.5% at RP_distal_] | 200 min  (70 min^****^) |

^*^Only items related to IMRT were extracted.

^**^Square brackets represent the values derived from clinical tolerances. All other listed tolerance values were determined empirically based on ±2SD from 5–10 consecutive measurements.

^***^Estimated time from preparation to completion of evaluation for each QA procedure.

^****^Actual working time of employees excluding waiting time and instrument analysis time.

Abbreviations: QA, quality assurance; IMRT, intensity-modulated radiotherapy; Au RR, gold reaction rate by conventional activation method

Table A3. Comparison of dosimetric monthly QA procedures in accelerator-BNCT with those in AAPM TG-142 and TG-198 report

| **Dosimetric QA procedures in AAPM TG-142 and TG-198 report^*^** | | |  | **Dosimetric QA procedures for accelerator-BNCT (*this study*)** | | |
| --- | --- | --- | --- | --- | --- | --- |
| **Procedure** | **Tolerance** | **Time required (range)** |  | **Procedure** | **Tolerance^**^** | **Time required**^***^ |
| ***Monthly QA*** |  |  |  | ***Monthly QA*** |  |  |
| Photon and Electron Output Constancy per beam | ±2% | 45–60 min |  | Charge Amount Monitor Calibration | ±1.0% | 30 min |
|  |  |  |  | Thermal/Epithermal Neutron Output Constancy with Charge Amount Monitor Adjustment (Au RR) | ±2.0% at RP_surface_ and RP_peak_; ±2.5% at RP_distal_ | 50 min |
|  |  |  |  | Gamma Output Constancy with Charge Amount Monitor Adjustment | ±7.0% at RP_surface_, RP_peak_, and RP_distal_  [±14.4% at RP_surface_; ±15.2% at RP_peak_; ±10.5% at RP_distal_] | 200 min  (70 min^****^) |
| Backup Monitor Chamber Constancy | (Omitted) | Included above |  | (No corresponding item  due to no backup monitor integrated) | | |
| Typical Dose Rate Output | (Omitted) | 10–15 min |  | (No corresponding item  due to single dose rate available) | | |
| Photon and Electron Beam Profile Constancy | ±1% | 10–60 min |  | Thermal/epithermal Neutron Beam Profile Constancy (Au RR) | ±2.0% from baseline | 220 min |
|  |  |  |  | Gamma Beam Profile Constancy | ±7.0% from baseline | 205 min  (75 min^****^) |
| Electron Beam Energy Constancy | (Omitted) | 20–30 min |  | (No corresponding item) | | |

^*^Only items related to IMRT were extracted.

^**^Square brackets represent the values derived from clinical tolerances. All other listed tolerance values were determined empirically based on ±2SD from 5–10 consecutive measurements.

^***^Estimated time from preparation to completion of evaluation for each QA procedure.

^****^Actual working time of employees excluding waiting time and instrument analysis time.

Abbreviations: QA, quality assurance; IMRT, intensity-modulated radiotherapy; Au RR, gold reaction rate by conventional activation method

Table A4. Comparison of dosimetric annual QA procedures in accelerator-BNCT with those in AAPM TG-142 and TG-198 report

| **Dosimetric QA procedures in AAPM TG-142 and TG-198 report^*^** | | |  | **Dosimetric QA procedures for accelerator-BNCT (*this study*)** | | |
| --- | --- | --- | --- | --- | --- | --- |
| **Procedure** | **Tolerance** | **Time required (range)** |  | **Procedure** | **Tolerance**^**^ | **Time required**^***^ |
| ***Annual QA*** |  |  |  | ***Annual QA*** |  |  |
| (No Corresponding Item) |  |  |  | Fast Neutron Output Constancy  (In RR) | (±10.5% at RP_surface_) | 1320 min  (110 min^****^) |
| Photon Flatness Change from Baseline | ±1% | 60–120 min |  | (No corresponding item  due to non-flatness beam) | | |
| Photon Symmetry Change from Baseline | ±1% | 60–120 min |  | Neutron/Epithermal Neutron Symmetry Change from Baseline (Au RR) | ±3.0% from baseline | 5 days  (1500 min^****^) |
|  |  |  |  | Gamma Symmetry Change from Baseline | ±7.0% from baseline | 450 min  (190 min^****^) |
| Electron Flatness Change from Baseline | (Omitted) | 60–120 min |  | (No corresponding item) | | |
| Electron Symmetry Change from Baseline | (Omitted) | 60–120 min |  | (No corresponding item) | | |
| Photon/Electron Output Calibration | (Omitted) | 120–180 min |  | (No corresponding item) | | |
| Spot Check of Field Size-Dependent Output Factors for Photon (2 or more field sizes) | (Omitted) | 30–60 min |  | (No corresponding item) | | |
| Output Factors for Electron Applicators (spot check of one applicator/energy) | (Omitted) | 60–90 min |  | (No corresponding item) | | |
| Photon Beam Quality (PDD_10_ or TMR_20:10_) | ±1% from baseline | 30–60 min |  | Neutron/Epithermal Neutron Beam Quality (Flux) | ±3.0% from baseline  [±3.7% at RP_surface_; ±3.1% at RP_peak_; ±3.2% at RP_distal_] | 400 min |
|  |  |  |  | Gamma Beam Quality | ±7.0% from baseline  [±14.4% at RP_surface_; ±15.2% at RP_peak_; ±10.5% at RP_distal_] | 210 min  (80 min^****^) |
| Electron Beam Quality (R_50_) | (Omitted) | 60–90 min |  | (No corresponding item) | | |
| Physical Wedge Transmission Factor Constancy | (Omitted) | 30–60 min |  | (No corresponding item) | | |
| Photon Monitor Unit Linearity (Output Constancy) | ±2% ≥ 5 MU ± 5% (2–4) MU, ±2% ≥ 5 MU ± 5% (2–4) MU, ±2% ≥ 5 MU | 30–60 min |  | Neutron Charge Amount Linearity (Au RR) | ±3.0% from a regression line | 1 week  (700 min^****^) |
| Electron Monitor Unit Linearity (Output Constancy) | (Omitted) | 30–60 min |  | (No corresponding item) | | |
| Photon Output Constancy vs Dose Rate | (Omitted) | 30–60 min |  | (No corresponding item) | | |
| Photon Output Constancy vs Gantry Angle | (Omitted) | 30–90 min |  | (No corresponding item) | | |
| Electron Output Constancy vs Gantry Angle | (Omitted) | 60–120 min |  | (No corresponding item) | | |
| Electron and Photon Off-Axis Factor Constancy vs Gantry Angle | (Omitted) | 60–120 min |  | (No corresponding item) | | |
| Arc Mode (expected MU, degrees) | (Omitted) | 15 min |  | (No corresponding item) | | |
| TBI/TSET Mode | (Omitted) | 5 min |  | (No corresponding item) | | |
| PDD or TMR and OAF Constancy measured at extended treatment distance | (Omitted) | 90–120 min |  | (No corresponding item) | | |
| TBI/TSET Output Calibration | (Omitted) | 90–120 min |  | (No corresponding item) | | |
| TBI/TSET Accessories | (Omitted) | 90–120 min |  | (No corresponding item) | | |

^*^Only items related to IMRT were extracted.

^**^Square brackets represent the values derived from clinical tolerances. All other listed tolerance values were determined empirically based on ±2SD from 5–10 consecutive measurements.

^***^Estimated time from preparation to completion of evaluation for each QA procedure.

^****^Actual working time of employees excluding waiting time and instrument analysis time.

Abbreviations: AAPM, the American Association of Physicists in Medicine; TG, Task Group; QA, quality assurance; IMRT, intensity-modulated radiotherapy; MU, monitor unit; TBI/TSET, total body irradiation/total skin electron therapy; PDD, percentage depth dose; TMR, tissue-maximum ratio; OAF, off-axis factor; In RR, reaction rate of indium by conventional activation method; Au RR, reaction rate of gold by conventional activation method
