# Supplementary material for: Determining a methodology of dosimetric quality assurance for commercially available accelerator-based boron neutron capture therapy system
Source: J Radiat Res. 2022 Jun 20;63(4):620–35. doi: 10.1093/jrr/rrac030 (PMC9303606; doi:10.1093/jrr/rrac030)
Supplement: SupplementaryData_flux_calculation_rrac030 [file supplementarydata_flux_calculation_rrac030.docx]

**Calculation of gold response rate**

If the reaction cross section of ^197^Au and neutron is *σ*, the neutron flux is *ϕ*, the decay constant of ^198^Au is *λ*, and the atomic numbers of ^197^Au and ^198^Au are *N_1_* and *N_2_*, respectively, then

$$\frac{dN_{1}}{dt}= -N_{1}\cdot\int_{0}^{\infty} \sigma_{E}\phi_{E}dE (1)$$

$$\frac{dN_{2}}{dt}= N_{1}\cdot\int_{0}^{\infty} \sigma_{E}\phi_{E}dE-\lambda N_{2} (2)$$

The activation reaction rate of ^197^Au is defined as:

$$RR=\int_{0}^{\infty} \sigma_{E}\phi_{E}dE (3)$$

Deriving *N_2_*(*t*) from equations (1), (2), and (3),

$$N_{2}\left( t \right)=\frac{N_{0}\cdot RR}{\lambda-RR}\left( e^{-RR\cdot t}-e^{-\lambda t} \right)$$

The radioactivity of ^198^Au, *A*(*t*) is

$$A\left( t \right)= \lambda N_{2}\left( t \right)$$

$$=\frac{\lambda N_{0}\cdot RR}{\lambda-RR}\left( e^{-RR\cdot t}-e^{-\lambda t} \right)$$

Usually, *λ*≫*RR*, *RR∙t*≪1 is satisfied, so

$$A\left( t \right)=N_{0}\cdot RR\left( 1-e^{-\lambda t} \right)$$

For the radioactivity produced per trend data of the accelerator (time of one interval of data, Δ*t*), the radioactivity of the i-th trend data, *A_i_*, expressed by the reaction rate of this trend in terms of *RR_i_*, is given by

$$A_{i}=N_{0}\cdot{RR}_{i}\left( 1-e^{-\lambda\Delta t} \right)$$

And if the reaction rate of the reference at the accelerator current of 1 C/sec is redefined as *RR*, then *A_i_* is derived using the accelerator charge in the trend, *Q_i_*, as follows:

$$A_{i}=N_{0}\cdot\frac{Q_{i}}{\Delta t}\cdot RR\left( 1-e^{-\lambda\Delta t} \right)$$

For this *A_i_*, the photon peak count, *Count_i_*, observed between the post-irradiation times *t_1_'* and *t_2_'* is defined by considering the decay constant *λ*, the detection efficiency *ε*, and the gamma-ray emission ratio *γ*:

$${Count}_{i}=\varepsilon\lambda\cdot\int_{t_{i1}’}^{t_{i2}’} A_{i}e^{-\lambda t}dt$$

If the time from the irradiation of the whole trend to the beginning of observation is *t_1_* and the time to the end of observation is *t_2_*, then

$t_{1}’=\left( n-i \right)\cdot\Delta t+t_{1}$, $t_{2}’=\left( n-i \right)\cdot\Delta t+t_{2}$

Therefore, the total photon peak counts, *C*, is

$$C=\sum_{i=1}^{n} {Count}_{i}$$

From this, the following equation can be derived:

$$RR=\frac{\lambda C}{\varepsilon\gamma N_{0}(e^{-\lambda t_{1}}-e^{-\lambda t_{2}})\sum_{i=1}^{n} \left[ \frac{Q_{i}}{\Delta t}\cdot\left( e^{-\lambda(n-i)\cdot\Delta t}-e^{-\lambda(n-i+1)\cdot\Delta t} \right) \right]}$$
